# The *GDF15* 3′ UTR Polymorphism rs1054564 Is Associated with Diabetes and Subclinical Atherosclerosis

**DOI:** 10.3390/ijms252211985

**Published:** 2024-11-07

**Authors:** Montse Guardiola, Josefa Girona, Emma Barroso, María García-Altares, Daiana Ibarretxe, Núria Plana, Josep Ribalta, Xavier Correig, Manuel Vázquez-Carrera, Lluís Masana, Ricardo Rodríguez-Calvo

**Affiliations:** 1 Research Unit on Lipids and Atherosclerosis, Universitat Rovira i Virgili, 43201 Reus, Spain; 2 Vascular Medicine and Metabolism Unit, “Sant Joan de Reus” University Hospital, 43204 Reus, Spain; 3 Pere Virgili Health Research Institute (IISPV), 43007 Tarragona, Spain; 4 Spanish Biomedical Research Centre in Diabetes and Associated Metabolic Disorders (CIBERDEM), Institute of Health Carlos III, 28029 Madrid, Spain; 5 Pharmacology Unit, Department of Pharmacology, Toxicology and Therapeutic Chemistry, Faculty of Pharmacy and Food Sciences, University of Barcelona, 08028 Barcelona, Spain; 6 Institut de Biomedicina de la Universidad de Barcelona (IBUB), University of Barcelona, 08028 Barcelona, Spain; 7 Institut de Recerca Sant Joan de Déu (IR-SJD), 08950 Barcelona, Spain; 8 Metabolomics Platform, Department of Electronic Engineering (DEEEA), Universitat Rovira i Virgili, 43007 Tarragona, Spain

**Keywords:** GDF15, rs1054564, diabetes, atherosclerosis

## Abstract

Growth differentiation factor 15 (GDF15) is a stress-response cytokine related to a wide variety of metabolic diseases. However, the impact of GDF15-specific genetic variants on the abovementioned conditions is poorly known. The aim of this study was to assess the impact of selected GDF15 single-nucleotide polymorphisms (SNPs) on metabolic disturbances and subclinical atherosclerosis. A cross-sectional study involving 153 participants of a metabolic patient-based cohort was performed. Three selected SNPs (rs888663, rs1054564 and rs1059369) in a locus on chromosome 19 including the *GDF15* gene were genotyped by Polymerase Chain Reaction (PCR), and its relationship with the serum GDF15 levels, health status and clinical variables were analyzed. Of the three SNPs analyzed, only rs1054564 showed different distributions between the healthy volunteers and patients suffering lipid alterations and associated disorders. Accordingly, just the rs1054564 variant carriers showed a significant increase in GDF15 serum levels compared to the wild-type carriers. The group of variant carriers showed a higher frequency of individuals with diabetes, compared to the wild-type carrier group, without showing differences in other metabolic conditions. Additionally, the frequency of individuals with atherosclerotic carotid plaque was higher in the rs1054564 variant carriers than in the wild-type carriers. Logistic regression models identified that the presence of the rs1054564 variant carriers increase the likelihood for both diabetes and carotid plaque independently of confounding factors. Overall, the findings of this study identify the rs1054564 variant as a potential indicator for the likelihood of diabetes and subclinical atherosclerosis.

## 1. Introduction

Growth differentiation factor 15 (GDF15), a divergent member of the transforming growth factor-β (TGF-β) superfamily [1,2,3,4], is a stress-response cytokine related to a wide variety of metabolic diseases, including type 2 diabetes mellitus (T2D) [5] and cardiovascular (CV) disease [6], among other conditions. Since both T2D and CV diseases share several common pathophysiological features [7,8], the common soil hypothesis postulated that both conditions share common genetic and environmental factors, which may explain the shared biomarkers for both conditions [9,10]. Among them, a proteomic study identified GDF15 as the highest-ranking protein associated with both T2D and coronary artery disease [11]. Additionally, GDF15 circulating levels have been proposed as a prognostic biomarker for insulin resistance and abnormal glucose control [12], and they have been found associated with T2D mortality [13]. Furthermore, GDF15 levels have been related to surrogate indicators of atherosclerosis and have been proposed as predictors of both all-cause and CV mortality [14,15,16,17]. Therefore, increasing evidence highlights the role of the GDF15 circulating levels as a relevant biomarker for both T2D and CV disease, beyond the well-established clinical risk factors for these conditions. Interestingly, genetic factors contribute to determine the GDF15 circulating concentrations similarly to cardiometabolic risk factors [18]. Nevertheless, the impact of *GDF15*-specific genetic variants on the abovementioned conditions has only been partially explored. Since *GDF15* expression has been associated with certain genetic single-nucleotide polymorphisms (SNPs) [18,19] and given that genetic factors may explain between 21.5 and 38% of the phenotypic variation in the GDF15 blood concentration [18,20], genetic *GDF15* variants may underlie the development of these pathologies.

The human *GDF15* gene is located on chromosome 19p13.11, containing two exons interspaced by one intron, rendering an mRNA product of 1200 base pairs coding for a 308-amino acid pro-protein [4]. Interestingly, two meta-analyses of genome-wide association studies (GWASs) have identified a genome-wide significant locus on chromosome 19, including the *GDF15* gene, which influences blood concentrations of GDF15 [18,20]. Of note, eight SNPs on this locus were independently identified in both studies [18,20]. Nevertheless, the complete picture of how these *GDF15* genetic variants impact on T2D and CVD has not been fully described.

Given the role of *GDF15* genetic variants in influencing its circulating levels, we hypothesize that *GDF15* genetic variants are associated with metabolic alterations. In this study, we analyzed the impact of selected *GDF15* SNPs on metabolic disturbances and subclinical atherosclerosis.

## 2. Results

### 2.1. Genotype and Allele Frequencies of GDF15 SNPs in Both Healthy Volunteers and Patients with Lipid Alterations and Related Disorders

The genotype of the three studied GDF15 SNPs (rs888663, rs1054564 and rs1059369) is shown in both healthy volunteers and patients (Table 1). Of the three SNPs studied, only rs1054564 showed significantly different distributions between the patients and healthy volunteers (*p* = 0.033). While among the healthy volunteers we found 78.6% wild carriers (GG) and 21.4% carriers of the variant (19.6% GC and 1.8% CC), this latter percentage rose to 38.1% among the patient group (37.1% GC and 1.0% CC). The allelic frequencies were similar to those already described (source https://www.ebi.ac.uk/gwas/ (accessed on 2 February 2024)).

### 2.2. Serum GDF15 Levels Are Increased in rs1054564 Variant Carriers

**Figure 1:**
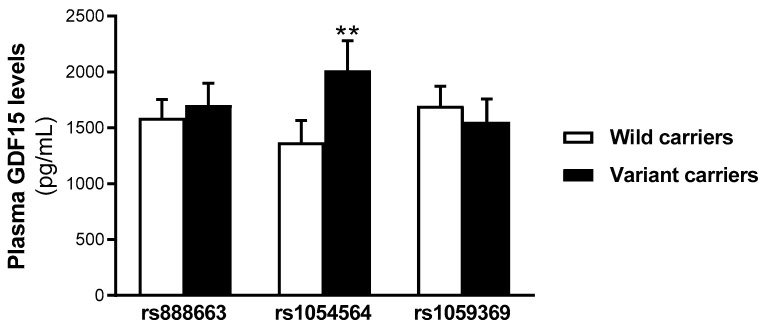
Serum GDF15 levels are increased in rs1054564 variant carriers compared to wild-type carriers. Data are expressed as the means ± SEM. ** *p* < 0.01 vs. rs1054564 wild-type carriers. *p* values are adjusted by age, glucose, insulin therapy, oral antidiabetic therapy and hypotensive therapy through the analysis of covariance (ANCOVA).

To explore whether the selected SNPs’ variant carriers influence the GDF15 circulating levels, the serum levels of this cytokine were determined. No statistically significant differences were found in the serum GDF15 levels between the wild-type and variant carriers for both the rs888663 and rs1059369 SNPs. However, the GDF15 levels were found increased in the serum from the rs1054564 variant carriers compared to the wild-type carriers (rs1054564 wild-type carriers (GG): 752.6 [463.6–1213.1] pg/mL; rs1054564 variant carriers (GC, CC): 1227.0 [704.6–1833.0] pg/mL, *p* = 0.002) (Figure 1).

### 2.3. Characteristics of Study Populations According to rs1054564 Genotype

The characteristics of the study populations according to the rs1054564 variants are shown in Table 2. Of the 153 individuals, 104 were rs1054564 wild-type carriers (GG), whereas 49 were variant carriers (47 GC and 2 CC). Both groups show similar age and gender distributions. The group of variant carriers showed a higher frequency of individuals with diabetes, compared to the wild-type carrier group (wild-type carriers: 38.5%; variant carriers: 63.3%, *p* < 0.004). No significant differences were found between the groups in other pathologic conditions, including hypertension, obesity, metabolic syndrome or liver steatosis. Accordingly, the glucose concentration was 16% higher in the group of rs1054564 variant carriers [119.0 (91.0–153.0) mg/dL] than in the rs1054564 wild-type carriers [102.2 (85.8–133.0) mg/dL], not showing significant differences in any of the other analyzed parameters.

In order to explore the prevalence of rs1054564 variants on diabetes, univariate and multivariate logistic binary regression models were performed. The presence of rs1054564 variant carriers increased the likelihood for diabetes both in the crude (OR 2.75 and 95% CI 1.37–5.56; *p* = 0.005) and adjusted models (Model 1: OR 2.98 and 95% CI 1.35–6.59; *p* = 0.007. Model 2: OR 3.13 and 95% CI 1.36–7.19; *p* = 0.007) (Table 3).

### 2.4. Association of the rs1054564 Variants with the Carotid Plaque Burden

**Figure 2:**
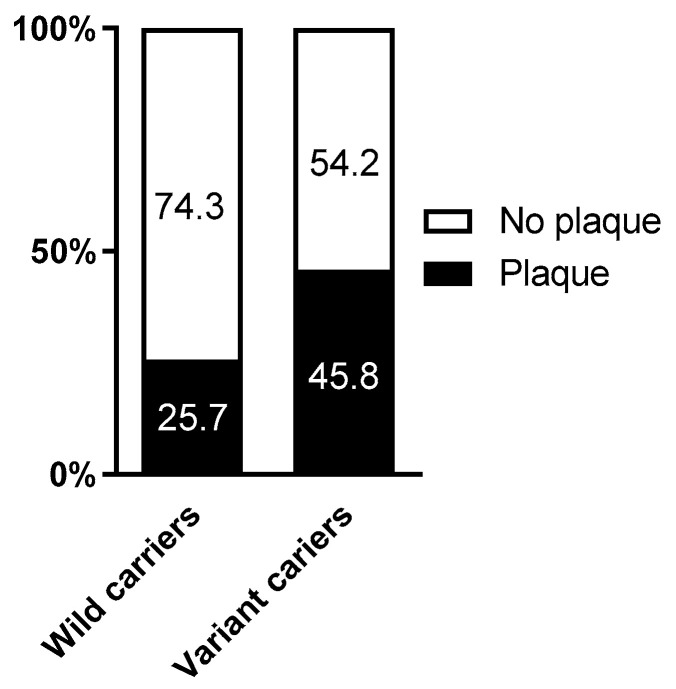
Percentage of plaque according to rs1054564. *p* = 0.014 between rs1054564 wild-type and variant carriers. Differences between groups were established by Chi-square tests.

Finally, we explored the frequency of individuals with atherosclerotic carotid plaque according to the rs1054564 variants. Whereas 45.8% of the rs1054564 variant carriers had developed atherosclerotic plaque, this was only observed in 25.7% of the wild-type carriers (*p* = 0.014) (Figure 2). The logistic regression models identified that the likelihood of atherosclerotic plaque was ~2.4-fold higher in the rs1054564 variant carriers than in the wild-type carriers (crude model: OR 2.44 and 95% CI 1.19–5.03; *p* = 0.015. Model 1: OR 2.44 and 95% CI 1.11–5.37; *p* = 0.026. Model 2: OR 2.41 and 95% CI 1.08–5.37; *p* = 0.032) (Table 4).

## 3. Discussion

Despite the well-known role of GDF15 in several (patho)physiological conditions [4], the influence of its genetic variants on these are still poorly studied. Given that certain genetic SNPs have been associated with *GDF15* expression [18,19] and its circulating levels may be influenced by genetic factors [18], we hypothesized that *GDF15* genetic variants may be associated with metabolic alterations. In this study, we explored the impact of selected SNPs from a chromosome 19 locus containing the *GDF15* gene [18,20] on metabolic disturbances and subclinical atherosclerosis.

Of the eight SNPs independently identified in two large GWASs [18,20], three selected SNPs (rs888663, rs1054564 and rs1059369) were chosen for genotyping. Of the three selected SNPs, only the rs1054564 variant carriers showed increased serum GDF15 levels compared to the wild-type carriers, without significant changes found in the GDF15 levels relative to the other two studied SNPs. These results conflict with previous studies suggesting that all these SNPs may significantly influence GDF15 blood concentrations [18,20]. However, our data cannot rule out the impact of these genetic variants (rs888663; rs1059369) on the blood concentrations of GDF15, probably due to the relatively small number of participants in our study cohort. Nevertheless, despite this limitation, the statistically significant increase in the serum GDF15 levels found in the rs1054564 variant carriers highlights the prominent role of this SNP in the regulation of GDF15 concentrations. Of note, previous studies have identified rs1054564 as the most significantly associated with circulating GDF15 levels among the SNPs near the 3′ untranslated region (UTR) of the *GDF15* locus [21,22]. The binding of miRNAs to 3′-UTR accelerates mRNA turnover. Therefore, genetic variants at the miRNA binding sites have a direct impact on miRNA-mRNA interactions, increasing mRNA stability and, therefore, protein translation. The rs1054564 contains binding sites for several miRNAs. Specifically, it has been previously reported that hsa-miR-1233-3p directly binds to rs1054564, and that the rs1054564-C allele partially abolishes the hsa-miR-1233-3p-mediated mRNA turnover and translational suppression of GDF15 [19]. Therefore, the increase in serum GDF15 levels found in the rs1054564 variant carriers in our population may be a consequence of the loss of translational repression of GDF15 by hsa-miR-1233-3p.

Because of the relevance of rs1054564 in the control of the GDF15 circulating levels, we classified our population into rs1054564 wild-type or variant carriers and analyzed its association with metabolic status in our population. The rs1054564 variant did not associate with hypertension, obesity, metabolic syndrome or liver steatosis. However, the percentage of diabetic patients was higher among carriers of the rs1054564 variant than in the wild-type group. Accordingly, the rs1054564 variant carriers showed higher glucose levels, without showing differences in any of the other analyzed variables. In line with these observations, the logistic regression models showed that the rs1054564 variant increased the likelihood of diabetes, even after adjusting for confounding factors. Our data are in line with a recent study reporting that the frequency of the rs1054564 variant carriers was significantly associated with an increased risk of T2DM compared to the wild-type genotype [23]. Therefore, the presence of the rs1054564 variant carriers may identify individuals at increased risk for diabetes. Additionally, given the role of GDF15 in atherosclerosis and CV disease [14,15,16], we analyzed the impact of the rs1054564 genetic variants on subclinical atherosclerosis. Our data revealed that in the group of variant carriers, there is a higher percentage of individuals with atherosclerotic plaque than in the wild-type group. Actually, the presence of the rs1054564 variant increases the likelihood of atherosclerotic plaque ~2.4-fold. To the best of our knowledge, this is the first study reporting a direct relationship between the rs1054564 variant carriers and atherosclerotic burden. Nevertheless, further studies are warranted in independent larger cohorts in order to confirm our data.

Despite no differences being found between the rs1054564 variant and wild-type carriers in any of the other studied variables, it is possible that the GDF15 influence on these is determined not just for rs1054564 but also for other parameters, both genetic and biochemical. However, the increase in GDF15 serum levels found in the rs1054564 variant carriers suggests that the impact of this SNP on diabetes and atherosclerosis may be due, at least partially, to the increase in GDF15 blood concentrations.

Our study has some limitations; some of them have already been mentioned. First, the relatively small sample size cannot rule out the impact of the rs888663 and rs1059369 variants on GDF15 blood concentrations. Additionally, it may attenuate the impact of the results found on the rs1054564 variant, and further studies in larger independent populations need to be carried out. Nevertheless, the relationships found among the rs1054564 variants and diabetes and carotid plaque are robust, despite the low number of participants in this study. Unfortunately, hemoglobin A1c (HbA1c) data were not available for all the participants in our cohort, so we do not have strict control of glycemic status. On the other hand, the cross-sectional nature of this study shows a fixed picture of the relationship between rs1054564 and the studied parameters at a given point and do not allow for exploring its impact over time. Additionally, it precludes establishing causal relationships between the rs1054564 and the studied outcomes. However, the increase in GDF15 serum level concentrations found in the rs1054564 variant carriers suggests that part of the observed effects may be mediated by the increase in the GDF15 blood concentrations.

## 4. Materials and Methods

### 4.1. Study Subjects

This cross-sectional study was performed in a subset of a well-characterized patient-based cohort attending the vascular medicine and metabolism unit of our university hospital [24,25]. Specifically, 98 individuals suffering lipid alterations and associated disorders (including obesity, metabolic syndrome, diabetes and cardiovascular disease) and 59 healthy volunteers free of metabolic disorders willing to participate were included in the study. Individuals with known serious diseases, including cancer, chronic lung, renal or liver disease, were excluded from this study [26]. Diabetes and metabolic syndrome were diagnosed according to the American Diabetes Association [27] and the Adult Treatment Panel III (ATPIII) criteria [28], respectively. Obesity [body mass index (BMI) ≥ 30 Kg/m^2^], liver steatosis [fatty liver index (FLI) ≥ 60 [29]] and arterial hypertension [systolic blood pressure ≥ 140 and/or diastolic blood pressure ≥ 90 mm Hg) [30]] were defined following standard clinical criteria.

Written informed consent was provided by all the study participants. This study was approved by the local Ethical and Clinical Investigation Committee according to the ethical standards outlined in the Declaration of Helsinki [31].

### 4.2. Clinical and Standard Biochemical Determinations

Anamnesis, anthropometric and physical examination data were recorded through standardized procedures. The information collected from the medical records includes age, gender, both systolic and diastolic blood pressure, weight, height and waist circumference, among other parameters. BMI was calculated from the weight and height measurements (Kg/m^2^).

Subclinical atherosclerosis was determined by performing carotid ultrasound imaging to determine the intima media thickness (IMT) of the right and left common carotid arteries by using a MyLab 60-X Vision sonographer (Esaote, Genova, Italy). The IMT value was calculated by averaging both carotid arteries. The presence of atherosclerotic plaque was defined as an IMT > 1.5 mm or focal structures into the arterial lumen that were 50% thicker than the surrounding IMT value [32].

The baseline serum samples were obtained from each participant via centrifugation from venous fasting blood samples, and aliquots were prepared for rapid storage at −80 °C in our center’s BioBank for further analysis. The cellular buffy coat was obtained, and the cells were stored at −80 °C for the DNA isolations and genotyping. Standard biochemical parameters [glucose, ultrasensitive C-reactive protein (usCRP), creatinine], lipids [total cholesterol, triglycerides and high-density lipoprotein cholesterol (HDLc)], transaminases [aspartate aminotransferase (AST) and alanine aminotransferase (ALT)] and gamma-glutamyl transpeptidase (GGT) were measured using colorimetric, enzymatic and immunoturbidimetric assays, respectively (Spinreact, SA, Spain; Wako Chemicals GmbH, Germany; and Polymedco, New York, NY, USA; CV < 4%), which were adapted to the Cobas Mira Plus Autoanalyser (Roche Diagnostics, Spain). The low-density lipoprotein cholesterol (LDLc) levels were calculated by the Friedewald formula: LDLc = total cholesterol − (HDLc + [triglycerides/5]). The acute phase glycoproteins Glyc-A and Glyc-B were assessed by nuclear magnetic resonance (1H-NMR) [33]. The serum GDF15 levels were determined in duplicate using a commercial sandwich enzyme-linked immunosorbent assay kit (Biovendor, Brno, Czech Republic; CV < 5%), following the manufacturer’s instructions.

Fatty liver and liver fibrosis were estimated by the FLI and Fibrosis-4 (FIB-4) algorithms, respectively [33].

### 4.3. SNP Selection and Genotyping

The *GDF15* SNPs were identified from the “Type 2 Diabetes Knowledge Portal” (http://type2diabetesgenetics.org (accessed on 3 September 2021)). Three selected SNPs (rs888663, rs1054564 and rs1059369) in the *GDF15* gene were chosen for genotyping according to the relatively high minor allele frequency (MAF), existence of previous reports supporting its association with metabolic disturbances or having functional evidence or being clinically relevant, by using the International HapMap database (http://hapmap.ncbi.nlm.nih.gov/ (accessed on 3 September 2021)) and the Clin Var (https://www.ncbi.nlm.nih.gov/clinvar/ (accessed on 3 September 2021)) tools.

The genomic DNA was isolated from the peripheral leukocytes from anticoagulated venous blood using the QIAamp DNA Blood Kit (Qiagen Iberia SL, Madrid, Spain) according to the manufacturer’s instructions. The selected SNPs were genotyped by Polymerase Chain Reaction (PCR) in an AbiPrism 7900HT Sequence Detection System (Applied Biosystems; Beverly, CA, USA). TaqMan SNP Genotyping assays-on-demand (Life Technologies; Madrid, Spain) were used for rs888663, rs1054564 and rs1059369 variant determination.

### 4.4. Statistical Analysis

The Kolmogorov–Smirnov test was used to determine the normality of the continuous variables. The data are expressed as frequencies for categorical variables, the mean ± SEM for continuous variables with normal distributions or the median and interquartile range for continuous variables with non-normal distributions. The differences between the groups were established by the Chi-squared (χ^2^) test for categorical variables; for continuous variables, Student’s *t* test or Mann–Whitney U were applied for normal and non-normal distributions, respectively. When appropriate, the differences between the groups were adjusted by confounding factors through the analysis of covariance (ANCOVA). Univariate and multivariate logistic binary regression models were performed for dichotomous variables to assess the likelihood of diabetes and atherosclerotic carotid plaque based on rs1054564. The results are presented as the odds ratio (OR) and 95% confidence interval (CI). The analyses were performed with the SPSS software (IBM SPSS Statistics, version 22.0). Statistically significant differences were considered with a two-sided *p* < 0.05.

## 5. Conclusions

Overall, our data identified the rs1054564 variants as potential indicators for the likelihood of diabetes and subclinical atherosclerosis.

## Figures and Tables

**Table 1:** Genotype frequencies of GDF15 SNPs in the study population.

SNPs	Healthy Volunteers	Patients	*p*-Value
rs888663			
TT	41 (73.2%)	69 (71.9%)	0.859
TG	12 (21.4%)	19 (18.8%)	
GG	3 (5.4%)	8 (8.3%)	
rs1054564			
GG	44 (78.6%)	60 (61.9%)	0.033
GC	11 (19.6%)	36 (37.1%)	
CC	1 (1.8%)	1 (1.0%)	
rs1059369			
TT	29 (51.8%)	59 (64.1%)	0.138
TA	24 (42.9%)	31 (33.7%)	
AA	3 (5.4%)	2 (2.2%)	

Data are shown as N (%). Differences in genotype distributions between groups were established by Chi-square tests.

**Table 2:** Baseline characteristics of the study population according to rs1054564.

	Wild-Type Carriers (N = 104)	Variant Carriers (N = 49)	*p*-Value
Clinical data			
Age (years)	55.2 ± 1.2	57.0 ± 1.5	0.374
Gender (F)	52.9%	51%	0.829
Hypertension	32.7%	42.9%	0.221
Diabetes	38.5%	63.3%	0.004
Obesity	32.7%	42.9%	0.221
Metabolic syndrome	53.8%	67.3%	0.114
Liver steatosis	43.7%	54.2%	0.230
Anthropometric and analytical data			
Systolic BP (mmHg)	130.3 ± 2.0	130.8 ± 2.8	0.887
Diastolic BP (mmHg)	78.0 (70.0–83.0)	78.3 (72.5–80.8)	0.863
Weight (Kg)	78.0 ± 1.5	80.1 ± 2.3	0.429
Waist circumference (cm)	96.8 ± 1.4	99.1 ± 2.2	0.354
BMI (Kg/m^2^)	28.6 ± 0.5	29.8 ± 0.8	0.175
Glucose (mg/dL)	102.2 (85.8–133.0)	119.0 (91.0–153.0)	0.040
Triglycerides (mmol/L)	1.4 (0.8–2.5)	1.6 (0.9–3.1)	0.390
Total cholesterol (mmol/L)	5.8 ± 0.1	6.1 ± 0.2	0.237
LDL (mmol/L)	3.5 ± 0.1	3.7 ± 0.2	0.358
HDL (mmol/L)	1.3 ± 0.0	1.3 ± 0.0	0.880
AST (U/L)	22.0 (20.0–26.0)	22.0 (20.0–30.0)	0.518
ALT (U/L)	16.0 (12.0–23.0)	19.0 (13.0–24.5)	0.310
GGT (U/L)	19.5 (14.0–30.8)	21.0 (14.0–38.5)	0.442
HsCRP (mg/L)	1.9 ± 0.1	2.1 ± 0.2	0.397
Glyc-A (µmol/L)	835.8 ± 28.9	908.2 ± 48.5	0.179
Glyc-B (µmol/L)	348.3 ± 7.4	361.8 ± 13.7	0.346
FIB-4	1.6 ± 0.0	1.6 ± 0.1	0.728
FLI (%)	43.1 (17.3–83.7)	68.9 (24.7–92.7)	0.136

Data are shown as percentage for categorical variables, mean ± SEM for continuous variables with normal distributions or median (interquartile range) for continuous variables with non-normal distributions. Normal distributions were analyzed by Student *t* test, non-normal distribution by U-Mann-Whitney and data gathered as categorical variables by Chi-square tests. Systolic BP: systolic blood pressure; Diastolic BP: diastolic blood pressure; BMI: body mass index; LDL: low-density lipoprotein; HDL: high-density lipoprotein; AST: aspartate aminotransferase; ALT: alanine aminotransferase; GGT: gamma-glutamyl transferase; HsCRP: high-sensitivity C-reactive protein; Glyc-A: glycoproteins A; Glyc-B: glycoproteins B; FLI: fatty liver index; FIB-4: fibrosis-4 score.

**Table 3:** Crude and adjusted models used to assess the association between the rs1054564 and presence of diabetes.

	OR (95% CI)	*p*
Crude	2.75 (1.37–5.56)	0.005
Model 1	3.13 (1.36–7.19)	0.007
Model 2	4.22 (1.57–11.34)	0.007

Logistic regression models (odds ratio, OR; 95% confidence interval, CI). Model 1 was adjusted by age and gender, and Model 2 was adjusted by age, gender and glucose (METHOD = Enter).

**Table 4:** Crude and adjusted models used to assess the association between the rs1054564 and presence of atherosclerotic plaque.

	OR (95% CI)	*p*
Crude	2.44 (1.19–5.03)	0.015
Model 1	2.44 (1.11–5.37)	0.026
Model 2	2.41 (1.08–5.37)	0.032

Logistic regression models (odds ratio, OR; 95% confidence interval, CI). Model 1 was adjusted by age, and Model 2 was adjusted by age and gender (METHOD = Enter).

## Data Availability

The data presented in this study will be provided by the corresponding author after reasonable inquiry.
